# Clinicopathological analysis of proliferative glomerulonephritis with monoclonal IgG deposits in 5 renal allografts

**DOI:** 10.1186/s12882-018-0969-3

**Published:** 2018-07-11

**Authors:** Jiqiu Wen, Wei Wang, Feng Xu, Jinsong Chen, Mingchao Zhang, Dongrui Cheng, Xuefeng Ni, Xue Li, Zhihong Liu

**Affiliations:** 1 National Clinical Research Center of Kidney Diseases, Jinling Hospital, Nanjing University Jinling School of Medicine, Nanjing, 210000 China; 2 National Clinical Research Center of Kidney Diseases, Jinling Hospital, Nanjing Medical University, Nanjing, 210000 China

**Keywords:** Bortezomib, Monoclonality, Kidney transplantation, PGNMID, Rituximab

## Abstract

**Background:**

We present a case series of 5 patients with proliferative glomerulonephritis with monoclonal IgG deposits (PGNMID) of renal allografts to better define its natural history, presenting characteristics, pathological features and treatment outcome.

**Results:**

These 5 patients presented 5 to 19 months post-kidney transplantation for complaints of serum creatinine (Scr) elevation, proteinuria or hematuria. Membranoproliferative glomerulonephritis (MPGN) pattern was the most frequently observed histological manifestation. Immunofluorescence showed monoclonal IgG3κin 3 patients and IgG3λ in the other 2 cases. Immunofluorescence staining helped to establish PGNMID in the absence of conspicuous microscopic changes in one case. Rituximab and bortezomib were effective in alleviating proteinuria in all 4 treated patients and decreasing Scr in 2 cases. Plasmapheresis treatment in another patient was not effective in preventing Scr elevation. At last-follow-up, 2 patients were in dialysis and 2 had improved kidney function with almost normal Scr and no proteinuria. The remaining one patient died of pulmonary infections.

**Conclusions:**

We conclude that PGNMID occurs early after kidney transplant. PGNMID should be included in the differential diagnoses of recurrent MPGN in renal allografts. Rituximab and bortezomib are helpful to decrease proteinuria and Scr in a subset of patients. Larger studies are needed to conclusively establish best treatment strategies for PGNMID in renal allografts.

## Background

One of the main conundrums complicating kidney transplantation (KT) is recurrence or de novo appearance of various glomerulonephritides. Lately a new distinct entity that recurs shortly after KT and microscopically predominantly manifests as membranoproliferative glomerulonephritis (MPGN) pattern and immunofluorescently defined as monoclonal IgG deposition in the mesangium and along the glomerular basement membrane (GBM) has been named proliferative glomerulonephritis with monoclonal IgG deposits (PGNMID) [1–3]. In fact, other light microscopic patterns, such as membranous features and endocapillary proliferative pattern have also been reported [1–3]. Although also caused by the deposition of monoclonal immunoglobulins, this entity is distinct from other common monoclonal immunoglobulin deposition disease, such as light- and/or heavy-chain deposition disease [4].

Some PGNMIDs cases were probably underrecognized previously, which may partially be explained by its rarity. As far as we are aware, only scarce case reports or small case series of PGNMID containing no more than 16 cases in the renal allografts have been reported to date [3, 5, 6]. Herein, we reported another case series containing 5 PGNMIDs that were referred to our center. We aim to better define the natural history, presenting characteristics, pathological features and treatment outcome of this elusive entity.

## Methods

Five patients with PGNMID were retrospectively identified after searching the electronic pathology report database at National Clinical Research Institute of Nephrology, Jingling Hospital (Nanjing, China) from January 2009 to April 2017. Pathological findings were carefully re-evaluated by two experienced renal pathologists (F.X, M.C.Z). Diagnostic criteria for PGNMID were in accordance with those described previously [1–3]. Briefly, immunofluorescence (IF) examination showed 1) positivity of IgG and negativity for IgA and IgM in only the glomeruli; 2) IgG heavy-chain subclass analysis must fulfill that only one subclass present; 3)predominantly one light-chain (kappa or lambda) positivity; 4)exclusion of cryoglobulinemia through clinical or laboratory workups. Additionally, light microscopic evidence of proliferative glomerulonephritis should also be present. All the 5 patients included were admitted for indication biopsies. Biopsy specimens were routinely processed by standard techniques for light microscopy (LM), IF and electron microscopy (EM) observations. Stains used for LM included HE, PAS, Masson and PASM stain. IF were performed with a panel of antibodies, including IgG, IgA, IgM, C1q, C4, kappa, lambda, C4d and IgG subclasses (IgG1, IgG2, IgG3 and IgG4).

Patients’ medical records were also reviewed for clinical and laboratory workup information. This study was approved by the Clinical Research Ethic Committee of Jingling Hospital. Written informed consents to publish their medical details were obtained from all the patients.

## Results

### Demographics, clinical features and laboratory workups

Demographics, clinical features and laboratory workups of 5 included cases were summarized in Table 1. Two patients have undergone native biopsy with an unequivocal diagnosis of MPGN while no clear diagnoses were made in the other 3 cases since no native biopsy have been performed. All renal allograft biopsies were indicative, including proteinuria (5 cases), elevated serum creatinine (Scr) (4 cases) and hematuria (5 cases). The time interval from transplant to PGNMID is generally short, ranging from 5 months to 19 months.

**Table 1:** Summary of demographics, clinical features, laboratory workups in 5 included patients

Patient	1		2			3	4	5	
	Pre-KT	Post-KT	1st post-KT biopsy	2nd post-KT biopsy	3rd post-KT biopsy	Post-KT	Post-KT	1st post-KT biopsy	2nd post-KT biopsy
Sex/Age	M/38	M/41	M/44	M/45	M/48	F/30	M/51	M/51	M/53
Cause of ERSD	MPGN (PGNMID)		MPGN			Unknown	Unknown	MPGN	
Transplant type		DCD	DCD			DCD	Living-related donor	DCD	
Immunosuppression protocol		Pre + MMF + CsA	Pre + MMF + FK506			Pre + MMF + FK506	Pre + MMF + FK506	Pre + MMF + FK506	
PRA		I-/II-	I-/II-	I-/II-	I-/II-	I+/II-	I-/II+	I-/II-	I-/II-
Time interval from KT to PGNMID		19 months		15 months^a^	52 months	10.5 months	11 months	5 months	33 months
Scr at biopsy (mg/dL)	9.2	0.77	1.26	1.24	3.05	1.2	1.67	1.88	1.33
Proteinuria at biopsy (g/24 h)	4+	2.21		2+	6.09	0.66	5.5	1.3	37.03
Hematuria at biopsy	2+	3+	ND	ND	2+	3+	4+	2+	3+
Urine C3 (mg/dL)	32.0	8.2	ND	ND	ND	ND	3	ND	ND
Serum C3/C4	ND	Normal	ND	ND	ND	Normal	Normal	Normal	Normal
Serum kappa/lambda	ND	ND	ND	Normal	Normal	4.2/2.51 mg/L	86.51/68.8 mg/L	Normal	ND
Cryoglobulin	Negative	Negative	ND	Negative	Negative	Negative	Negative	Negative	ND
C3NeF	Negative	Negative	ND	ND	ND	Negative	Negative	Negative	ND
CFH	ND	ND	ND	ND	ND	Normal	Normal	Negative	ND
CFH antibody	ND	Negative	ND	ND	ND	Negative	ND	Negative	ND
HBV/HCV/HIV	Negative	Negative	Negative	Negative	Negative	Negative	Negative	HCV+	Negative
Serum M spike	ND	ND	ND	Negative	Negative	Negative	Negative	Negative	Negative
Bone marrow aspirate	ND	ND	ND	ND	ND	Negative	Negative	Negative	Negative

Range: urine C3 < 2.76 mg/dl; serum kappa: 7-23 mg/L; serum lambda: 6-20 mg/L

*Abbreviations*: *C3NeF* C3 nephrotic factor, *CFH* complement factor H, *CsA* cyclosporin, *KT* kidney transplantation, *MMF* mycophenolate mofetil, *MPGN* membranoproliferative glomerulonephritis, *ND* not done or not available, *PRA* panel reactive antibody, *Pre* predisone, *Scr* serum creatinine

^a^: in the 2nd biopsy of case 2, PGNMID was retrospectively diagnosed. The diagnosis at that time was mild mesangial proliferation

Laboratory work-ups indicated normal serum complement C3/C4 levels in all 4 cases tested. Nevertheless, urine C3 excretion was increased in all 2 cases measured. Serum light-chain levels varied in different patients. Laboratory testing for cryoglobulin, C3 nephritic factor, complement factor H, complement factor H antibody and HBV/HCV/HIV were all negative except in case 5 in which HCV was present. These tests help to exclude cryoglobulinemia, C3 glomerulopathy and immune-complex-mediated glomerulonephritis that commonly manifested as MPGN pattern histopathologically. Serum monoclonal protein spike as detected by electrophoresis was negative in all 4 cases tested and bone marrow aspirate examination was also negative for plasma cell dyscrasias in 3 performed cases.

### Renal biopsy findings

Renal pathological findings of a total of 9 biopsies were summarized in Table 2.

**Table 2:** Summary of renal pathology findings of 5 included patients

Patient	1		2			3	4	5	
	Pre-KT	Post-KT	1st post-KT biopsy	2nd post-KT biopsy*	3rd post-KT biopsy	Post-KT	Post-KT	1st post-KT biopsy	2nd post-KT biopsy
Microscopic pattern	MPGN	MPGN	Mild mesangial proliferation	Mild mesangial proliferation	MPGN + Crescents	Moderate to severe mesangial proliferation	Diffuse proliferation	Moderate mesangial proliferation	MPGN
Glomeruli sclerosed (%)	24	0	7.6	0	26.7	0	27.8	0	5.3
Crescents (%)	23.8	16.7	0	0	53.3	0	0	3	10.5
IF/TA	Moderate	Mild	No IF/TA	No IF/TA	moderate	Mild	Moderate	Mild	Moderate
IgG isotype	IgG3/kappa	IgG3/kappa	None	IgG3/kappa	IgG3/kappa	IgG3/Lambda	IgG3/Lambda	IgG3/kappa	IgG3/kappa
C3	++	++	–	++	–	+++	++	++	++
C1q	++	+	–	–	+	+++	++	+	++
Electron-dense deposits	GBM + mesangial	GBM + mesangial	Negative	Mainly mesangial	Subendothelial + mesangial	Subendothelial + mesangial + subepithelial	GBM	GBM + mesangial	GBM + mesangial

*Abbreviations*: *AAMR* acute antibody-mediated rejection, *GBM* glomerular basement membrane, *IF/TA* interstitial fibrosis and tubular atrophy, *KT* kidney transplantation, *MPGN* membranoproliferative glomerulonephritis, *PGNMID* proliferative glomerulonephritis with monoclonal IgG deposits

*: in the 2nd biopsy of case 2, PGNMID was retrospectively diagnosed. The diagnosis at that time was mild mesangial proliferation

**Fig. 1:**
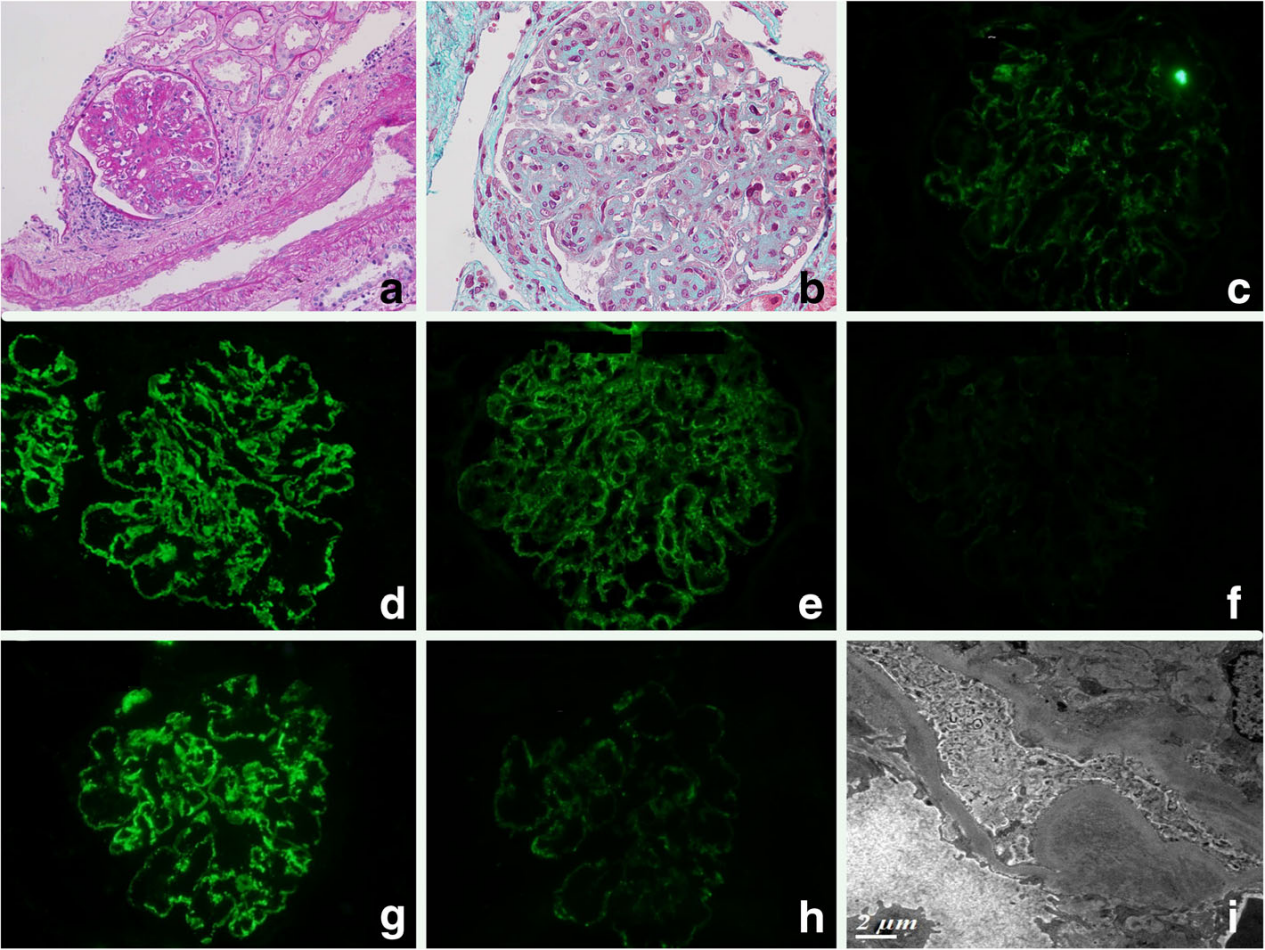
Renal biopsy findings in the renal allograft of Patient 1. **a** Classic MPGN-pattern is obvious in this allograft biopsy (PAS × 200). **b** Masson staining showed a few fuchsinophilic immune-complexes. **c-h** IF profiles: positive IgG (**c**), IgG3 (**d**), C3 (**g**), C1q (**h**), kappa (**e**) and negative lambda (**f**) staining that were similar to the native kidney biopsy IF results. **i** EM showed electron-dense deposits along the GBM

Patient 1: Microscopic observation of the native kidney biopsy showed a classic MPGN pattern with mesangial proliferation leading to hypercellularity and glomerular tuft lobulation. Mesangial interposition resulting in the so-called characteristic “tram tracks” or “double contours” could also be frequently observed and validated by silver stain. Masson trichrome stain revealed intense fuchsinophilic immune-complexes in the mesangium and along the GBM. IF indicated intense IgG, C3 and C1q positivity in the mesangium and along the GBM with granular texture. Heavy-chain subclass analysis showed only IgG3 positivity with negativity for IgG1, IgG2 and IgG4. Light-chain IF demonstrated kappa positivity and lambda negativity. EM findings were congruent with IF with electron-dense deposits along the GBM and in the mesangium. PGNMID recurrence was documented with the allograft biopsy showed similar LM (Fig. 1a, b) and IF features (Fig. 1c-h) with the native kidney biopsy, even though fuchsinophilic deposits observed by Masson trichrome stain and EM (Fig. 1i) were less conspicuous and widespread.

**Fig. 2:**
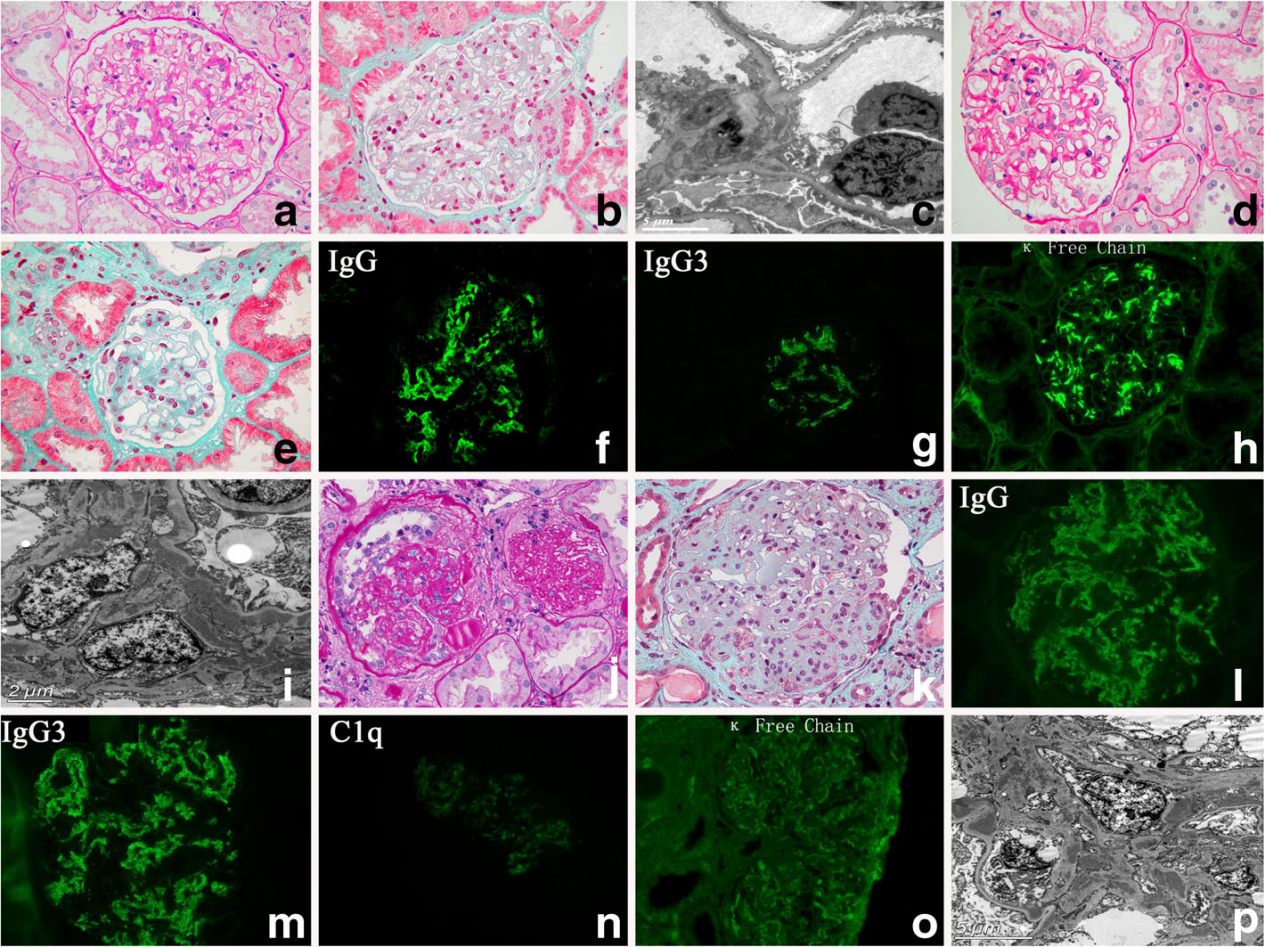
Pathological findings of 3 consecutive renal allograft biopsies in Patient 2. In the 1st allograft biopsy, only mild mesangial proliferation were seen (**a**, PAS × 400). No fuchsinophilic immune-complexes were found (**b**, Masson× 400). EM was normal with no obvious electron-dense deposits noticed (**c**). In the 2nd allograft biopsy that was performed 12 months later, LM only revealed mild to moderate mesangial proliferation (**d**, PAS × 400). Still, no fuchsinophilic immune-complexes could be observed (**e**, Masson× 400). Nevertheless, IF showed bright granular staining for IgG (subcalss IgG3) and kappa predominantly in the mesangium (**f-h**). EM revealed obviously electron-dense deposits mainly in the mesangium (**i**). A 3rd biopsy performed 36 months after the 2nd biopsy demonstrated MPGN pattern and crescents (**j**, PAS × 400) and obvious fuchsinophilic immune-complexes (**k**, Masson× 400). In addition to similar IF findings with the 2nd biopsy, positive staining for C1q was also present (**l-o**). EM (**p**) showed extensive electron-dense deposits in the subendothelium and mesangium

Patient 2: Medical record documented the native kidney disease was MPGN, however, a detailed re-evaluation of the slides of the native kidney biopsy was unobtainable. In the 1st allograft biopsy, only mild mesangial proliferation were seen (Fig. 2a-b) with negative IF and EM (Fig. 2c). In the 2nd allograft biopsy that was performed 12 months later, LM also only revealed mild mesangial proliferation (Fig. 2d-e). Nevertheless, IF showed bright granular staining for IgG (subcalss IgG3) and kappa predominantly in the mesangium(Fig. 2f-h). IF staining for C3, C1q and IgA, IgM were all negative. EM revealed obviously electron-dense deposits mainly in the mesangium (Fig. 2i). A 3rd biopsy performed 36 months after the 2nd biopsy demonstrated MPGN pattern in LM (Fig. 2j-k). Crescents and moderate interstitial fibrosis and tubular atrophy were also seen. Fuchsinophilic immune-complex deposits can occasionally be seen in the mesangium. In addition to similar IF findings with the 2nd biopsy, positive staining for C1q was also present (Fig. 2l-o). EM (Fig. 2p) showed extensive electron-dense deposits in the subendothelium and mesangium.

Patient 3: This patient was complicated by acute antibody-mediated rejection as indicated by extensive peritubular capillaritis and extensive peritubular capillary C4d positive staining. LM showed diffuse proliferative glomerulonephritis. IF were typical of PGNMID with monoclonal IgG3 and lambda positivity. C3 and C1q staining were also positive. EM demonstrated extensive electron-dense deposits in the mesangium and in the subendothelial spaces. Subepithelial electron-dense deposits were also occasionally observed.

Patient 4: LM and IF features were similar to those found in patient 3. Nonetheless, EM showed diffuse electron-dense deposits along the GBM. Widespread foot process effacement was seen, which explained massive proteinuria in this patient.

Patient 5: The 1st allograft biopsy was noticeable for obvious fuchsinophilic immune-complexes in the mesangium. IF indicated monoclonality of IgG3. Tubulitis was also present, which was consistent with acute cellular rejection. In the 2nd allograft biopsy 28 months later, extensive MPGN-pattern was observed in the majority of glomeruli. Histological deterioration as indicated by increasing interstitial fibrosis, glomerular lobulation and widespread foot process effacement were observed.

### Treatment response, adverse events and prognosis

Treatment regimens, treatment-related adverse events and follow-up results were summarized in Table 3. PGNMID was treated with plasma exchange in 2 patient (patient 1 and 5), rituximab in 1 patient (patient 2) and bortezomib in 3 cases (patient 3, 4 and 5). Patient 3 was also treated with pulse steroid for concomitant antibody-mediated rejection. Patient 5 was first treated with sofosbuvir and daclatasvir for HCV infection, then with pulse steroid since acute rejection was considered to be the main cause for Scr elevation. No serious treatment-related complications were observed except varicella infection in patient 4. Long-term follow-up in patient 2 and 3 showed worsening Scr, resulting in dialysis. Scr and proteinuria in patient 4 and 5 decreased and then stabilized. Patient 1 died of pulmonary infections at 13 months follow-up. After treating with bortezomib and plasma exchange, patient 5 experienced dramatically decreased proteinuria.

**Table 3:** Treatment protocols and follow-up in 5 included patients

Patient	1	2			3	4	5	
	Post-KT	1st post-KT biopsy	2nd post-KT biopsy	3rd post-KT biopsy	Post-KT	Post-KT	1st post-KT biopsy	2nd post-KT biopsy
Scr/proteinuria at biopsy	0.77/2.21			3.05/6.09	1.2/0.66	1.67/5.5		1.33/37.03
Treatment	Plasma exchange	None	None	Rituximab	Pulse steroid + bortezomib	Bortezomib	Sofosbuvir + Daclatasvir + pulse steroid	Bortezomib + plasma exchange
Treatment-related complication	None			None	None	Varicella infection	None	None
Follow-up time^a^	13 months			39 months	7 months	11 months		1 months
Last follow-up Scr(mg/dl)/Proteinuria(g/d)	Death due to pulmonary infections			In dialysis	In dialysis	1.1/−		1.25/14

^a^: follow-up time here denotes the time interval between treatment to last outpatient follow-up

## Discussion

PGNMID is a rare disease entity that was first reported by Nasr et al. [1] in the native kidneys mimicking kidney lesions caused by immune-complex-mediated glomerulonephritis. To the best of our knowledge, the largest series reported in the native kidneys contained 60 cases [7] and only 16 cases [5] of recurrent or de novo PGNMID have been reported in renal allografts. In this retrospective study, some observations that we have made regarding PGNMID are of important clinical significance. First, PGNMID generally occurs early after KT. The longest time interval from KT to PGNMID occurrence in our series is 19 months. Second, PGNMID is rare; however, its IF manifestation is stereotypical with solely positivity of one subclass of IgG, one subclass of light-chains and frequently positive C3 and C1q staining in only the glomeruli. Third, the gradual progression of PGNMID, as illustrated by case 2, is characterized by positive IF staining for IgG subclass and EM finding of electron-dense immune-complex which is unappreciable by LM then to apparent mesangial expansion and immune-complex deposition as detected by Masson staining. At last, rituximab and bortezomib, two drugs that function to eliminate B lymphocytes and plasma cells respectively, were effective in a subset of patients included in our study.

The clinical manifestations and laboratory workups of de novo or recurrent PGNMID are totally non-specific. Frequent presenting complaints included elevated Scr, proteinuria and hematuria. Nasr et al. [2] reported that 97.1 and 77.1% of patients had > 1 g/24 h proteinuria and hematuria respectively at the time of biopsy. Interestingly, PGNMID was incidentally found in case 3 and 5 when biopsied for suspicion of acute rejection. In fact, it has been reported that PGNMID was concomitantly found in biopsy specimens that were also present for other common conditions, such as BK-virus nephropathy and transplant glomerulopathy [6]. Laboratory workups for common etiologies of MPGN are frequently negative, including cryoglobulinemia, factors leading to complement alternative pathway dysregulation (C3 nephritic factor, complement factor H antibody) and infectious etiologies. Despite the glomerular deposits are monoclonal in nature, serum monoclonal protein (M spike) and bone marrow aspirate examination are all negative in cases tested. No serum or urine parapprotein were detected in all the patients tested in the renal allograft patients reported by Nasr et al. [3]. However, serum or urine parapprotein and plasma cell dyscrasia was detected in 29.7 and 9% of patients respectively in the largest cohort of native kidneys [7]. Due to potent immunosuppression, detecting an overt lymphoproliferative disorder is even more difficult in transplant patients. Nonetheless, overt plasma cell neoplasia has been reported in a patient with renal allograft [8]. Due to our limited follow-up time and incomplete work-up for lymphoproliferative disorders, it is prudent to follow-up these patients closely for early detection.

The microscopic manifestations of PGNMID in the renal allografts are almost the same as those in the native kidneys [2, 3]. MPGN-like pattern and diffuse proliferative glomerulonephritis-like pattern are predominantly seen in this series. The key distinguishing and diagnostic feature of PGNMID is by IF with demonstration of monoclonality of both heavy and light chain with both C3 and C1q frequently being positive. EM is not only helpful in corroborating IF findings, but also conducive for excluding other common mimickers, such as immunotactoid glomerulopathy, fibrillary glomerulonephritis and LHCDD. In the transplant biopsy specimens, transplant glomerulopathy should also be excluded since it may also show MPGN-like pattern. The mere diagnosis of MPGN or MPGN-like changes has now been strongly discouraged since this diagnosis provides little information regarding the underlying etiology and thus unhelpful to clinical management and recurrence risk assessment [9]. The proposed main diagnostic modality has now switched to IF [10]. A combined use of LM, IF and EM is strongly favored as long as the patient condition and specimen volume permits. It is obvious that PGNMID will be missed when IF is omitted with only LM performed as illustrated in case 2 of our series.

Given that PGNMID occurs shortly after KT, this risk should be discussed with patients before transplant. It is unequivocal that PGNMID was recurrent in patient 1. Recurrence was strongly favored in patient 2 even though no original native kidney biopsy material can be re-evaluated. Similarly, de novo appearance of PGNMID in case 3, case 4 and case 5 were highly unlikely given that these 3 patients presented shortly after KT. In a protocol biopsy study, Nasr and associates [3] demonstrated that PGNMID recurrence can be documented at biopsy as early as a mean of 3.8 months post-KT.

The exact etiology and pathogenesis of PGNMID remains largely unknown, rendering correspondingly etiology-based treatment unpractical to date. All the implicating IgG subtype in our series is IgG3, which is consistent with findings reported in both the native and transplanted kidneys [2, 3]. Many investigators attributed this finding to the speculation that IgG3 subtype has strong affinity to the glomeruli due to its propensity to self-aggregate and binds to the GBM which is negatively-charged and fixes complement, causing endothelial injury and ensuing endocapillary and mesangial proliferation [1–3]. Nevertheless, serum complement levels were normal in our series in most patients, as is often the case reported by others [1–3, 11]. Lately there were reports [12, 13] that PGNMID was a renal manifestation of monoclonal gammopathy of renal significance which is characterized by renal damage caused by the deposition of monoclonal immunoglobulins secreted by low-grade lymphoproliferative disorders. Thus a complete hematologic work-up including serum and urinary M protein detection, bone marrow aspirate and/or bone marrow biopsy, serum light-chain measurement or even fluorescence in situ hybridization for multiple myeloma should be performed to exclude underlying hematologic dyscrasias.

Treatment strategy employing rituximab or bortezomib, though justified theoretically to reduce immunoglobulin levels and thus alleviate glomerular injury, failed to be demonstrated to be effective in decreasing Scr in 2 of the 4 treated patients. Nevertheless, rituximab and bortezomib are effective in alleviating proteinuria in all 4 patients. Plasmapheresis treatment alone failed to avert Scr elevation in patient 1. In the native kidneys, Nasr group [3] reported that among 4 patients treated with rituximab, 2 patients achieved partial remission as defined as 50% reduction of proteinuria. This group also found favorable effect of rituximab in alleviating proteinuria in allografts with PGNMID. The experience of bortezomib use in PGNMID is limited. It has been found to be ineffective in alleviating proteinuria or stabilizing renal function in a patient with PGNMID in the native kidneys [2]. In another report [5], carfilzomib, also a protease-inhibitor like bortezomib, is effective in reducing Scr in a renal allograft with PGNMID. There is a case report [14] observing that plasmapheresis combined with intravenous immunoglobulins and mycophenolate mofetil reduced Scr and proteinuria from 2 mg/dl and 3.3 g/24 h to 1.1 mg/dl and 0.2 g/24 h respectively. To date, there is no proven effective therapeutic approaches for PGNMID and the number of treated patients is small. Therefore, larger studies are needed to conclusively demonstrate the efficacy of various treatment strategies.

## Conclusions

In conclusion, we reported 5 rare cases of PGNMID in the renal allografts that presented shortly after KT. The diagnosis of PGNMID relies heavily on IF, which could detect monoclonal IgG deposits even in the absence of obvious LM morphological changes. Rituximab and bortezomib are helpful for alleviating proteinuria and reducing Scr in a subset of patients. Due to limited case number and retrospective nature of our report, larger prospective studies are needed to conclusively provide evidence-based treatment strategies and prognosticators.

## Abbreviations

EM: Electron microscopy
GBM: Glomerular basement membrane
IF: Immunofluorescence
KT: Kidney transplantation
LM: Light microscopy
MPGN: Membranoproliferative glomerulonephritis
PGNMID: Proliferative glomerulonephritis with monoclonal IgG deposits
Scr: Serum creatinine
